# Supplementary material for: Effect of an educational program on the knowledge, attitudes, and practices of hospital nurses regarding evidence-based practice: A quasi-experimental study
Source: PLoS One. 2026 Apr 15;21(4):e0347252. doi: 10.1371/journal.pone.0347252 (PMC13082721; doi:10.1371/journal.pone.0347252)
Supplement: Attachment — Submitted filename: Review Report.pdf [file pone.0347252.s002.pdf]

Manuscript

Effect of an educational program on the knowledge, attitudes, and practices of hospital nurses regarding evidence-based practice: A quasi- experimental study

Feedback for the author(s)

Major Comments

1. Study Design and Internal Validity

- The quasi-experimental design with hospital-level group allocation is appropriate given contextual constraints; however, non-random assignment of hospitals introduces a substantial risk of selection bias and contamination.
- Although baseline characteristics are statistically comparable, hospital-level differences (organizational culture, leadership, workload) may still explain part of the observed effects.

Recommendation:
The authors should

- Explicitly acknowledge cluster-level confounding as a limitation.
- Clarify whether any steps were taken to minimize contamination (e.g., staff movement, informal knowledge sharing between hospitals).

2. Sampling Strategy and Generalizability

- Convenience sampling was used, and only bachelor-prepared nurses were included.
- The sample is limited to four governmental hospitals in one city.

Recommendation:
The authors should strengthen the limitations section by clearly stating that:

- Findings may not generalize to diploma nurses, private hospitals, or other regions of Iraq.
- Volunteer bias may have inflated intervention effects due to higher motivation among participants.

3. Statistical Results and Effect Sizes

- The reported effect sizes are exceptionally large (e.g., $\eta^2 = 0.85$ for knowledge).
- Such magnitudes are uncommon in educational interventions and raise concerns about: Measurement sensitivity, Response bias and Ceiling effects

Recommendation:

The authors should:

- Discuss why such large effects were observed.

- Acknowledge the possibility of social desirability bias, especially in self-reported practice scores.
- Consider adding confidence intervals for effect sizes or interpreting them more cautiously.

4. Self- Reporting Bias

- The "Practice" domain relies on a self-administered questionnaire. Nurses may over-report "always" performing EBP steps due to social desirability. This is a significant limitation.

Recommendation:
The discussion should clearly state that:

- Improvements in practice reflect perceived or reported behavior, not verified clinical behavior.
- Future research should incorporate audits, observations, or patient-level outcomes.

Minor Comments

1. **Abstract**
   - Consider adding the **study design (quasi-experimental)** explicitly in the first line.
   - Clarify that practices are **self-reported.**
2. **Introduction**
   - While the gap in Iraq is noted, the authors should briefly specify if there are any national nursing policies in Iraq that mandate EBP, which would further strengthen the **"need"** of this study.
3. **Methods**
   - Specify whether the educational sessions were delivered by the same instructor.
   - Clarify attendance thresholds (e.g., minimum sessions required for inclusion)
4. **Ethics**
   - Verbal consent is acceptable but briefly justify why written consent was not obtained.
