# Supplementary material for: Effect of an educational program on the knowledge, attitudes, and practices of hospital nurses regarding evidence-based practice: A quasi-experimental study
Source: PLoS One. 2026 Apr 15;21(4):e0347252. doi: 10.1371/journal.pone.0347252 (PMC13082721; doi:10.1371/journal.pone.0347252)
Supplement: Attachment — Submitted filename: Response_to_Reviewers_auresp_2.docx [file pone.0347252.s005.docx]

**Response to Reviewers**

**Journal Requirements:**

**1. We note that there is identifying data in the Supporting Information file <S1_Dataset.xlsx>. Prior to sharing human research participant data, authors should consult with an ethics committee to ensure data are shared in accordance with participant consent and all applicable local laws.
Please remove or anonymize all personal information (age), ensure that the data shared are in accordance with participant consent, and re-upload a fully anonymized data set. Please note that spreadsheet columns with personal information must be removed and not hidden as all hidden columns will appear in the published file.**

**Response:**

Thank you for your comment. All identifiable data have been carefully reviewed and removed from the dataset. Any variables that could directly or indirectly identify participants have been deleted or replaced with non-identifiable sequential numbers. Specifically, the age column was removed, and years of experience were converted into categorical variables to minimize the risk of participant identification while preserving data utility.

The revised dataset is now fully anonymized and complies with ethical standards and data-sharing policies.

**2. If the reviewer comments include a recommendation to cite specific previously published works, please review and evaluate these publications to determine whether they are relevant and should be cited. There is no requirement to cite these works unless the editor has indicated otherwise.**

**Response:**
We confirm that the reviewer did not recommend citing any specific previously published works.
 **3. Please review your reference list to ensure that it is complete and correct. If you have cited papers that have been retracted, please include the rationale for doing so in the manuscript text, or remove these references and replace them with relevant current references. Any changes to the reference list should be mentioned in the rebuttal letter that accompanies your revised manuscript. If you need to cite a retracted article, indicate the article’s retracted status in the References list and also include a citation and full reference for the retraction notice.**

**Response:**
We have carefully reviewed the entire reference list to ensure its completeness and accuracy. All cited articles were checked for retraction status, and none were found to be retracted. Therefore, no changes to the reference list were necessary in this regard.

**Reviewer comments:**

**Major Comments**

**1. Interpretation of Effect Sizes**

The authors have provided a reasonable explanation for the large effect sizes observed, particularly for knowledge (η² = 0.85). However, the interpretation could be further strengthened by adopting a slightly more cautious tone.

It is recommended to explicitly acknowledge that such unusually large effect sizes may also reflect factors such as measurement alignment with the intervention, limited variability, and potential response bias, rather than purely the magnitude of the intervention effect. Adding one or two sentences of evidence that emphasize this caution would improve interpretative rigor.

**Response:**
We thank the reviewer for this suggestion. We have revised the Discussion to note that the large effect size for knowledge (η² = 0.85) should be interpreted with caution, as it may reflect not only the intervention but also low baseline knowledge, close alignment of the outcome measure, quasi-experimental design, cluster-level influences, reliance on self-reported measures, potential response bias, and limited within-group variability.

**Minor Comments**

1. **Language and Style**

• Minor grammatical refinement is recommended (e.g., replace “At the other hand” with “On

the other hand”).

. A few sentences in the Discussion section could be shortened for clarity.

**Response:**

- We appreciate the reviewer’s attention to language and style. The manuscript has been carefully revised to correct minor grammatical issues throughout the manuscript.
- We thank the reviewer for this suggestion. The Discussion section has been carefully revised to shorten several long sentences for improved clarity and readability, while ensuring that all key information and interpretations are fully retained **(Page 17-18, Line 310-311; 314).**

2. **Clarity in Discussion**

• Some sections of the Discussion are slightly dense; light editing for readability would enhance overall flow.

**Response:**

We appreciate the reviewer’s feedback. The Discussion sections and limitation has been carefully edited to improve readability and flow. Long or dense sentences have been simplified, and paragraphs have been adjusted for clarity, while ensuring that all key findings and interpretations are fully preserved **(Page 17-19, Line 328-335; 344-351; 367-379).**
