# Supplementary material for: Effect of an educational program on the knowledge, attitudes, and practices of hospital nurses regarding evidence-based practice: A quasi-experimental study
Source: PLoS One. 2026 Apr 15;21(4):e0347252. doi: 10.1371/journal.pone.0347252 (PMC13082721; doi:10.1371/journal.pone.0347252)
Supplement: Attachment — Submitted filename: Reviewer Comments to the Author.pdf [file pone.0347252.s004.pdf]

Reviewer Comments to the Author

The authors have made substantial revisions in response to the previous review. The manuscript has improved considerably in clarity, methodological transparency, and balance of interpretation. The study addresses an important and under-researched area and provides valuable context-specific evidence from Iraq.

Overall, the manuscript is methodologically sound, and the conclusions are generally supported by the data. Most of the major and minor concerns raised in the previous round have been adequately addressed. However, a few minor issues remain that should be addressed before the manuscript can be accepted.

Major Comments

1. Interpretation of Effect Sizes

The authors have provided a reasonable explanation for the large effect sizes observed, particularly for knowledge ($\eta^2 = 0.85$). However, the interpretation could be further strengthened by adopting a slightly more cautious tone.

It is recommended to explicitly acknowledge that such unusually large effect sizes may also reflect factors such as measurement alignment with the intervention, limited variability, and potential response bias, rather than purely the magnitude of the intervention effect. Adding one or two sentences of evidence that emphasize this caution would improve interpretative rigor.

Minor Comments

1. **Language and Style**

- Minor grammatical refinement is recommended (e.g., replace "At the other hand" with "On the other hand").
- A few sentences in the Discussion section could be shortened for clarity.

2. **Clarity in Discussion**

- Some sections of the Discussion are slightly dense; light editing for readability would enhance overall flow.

Overall Evaluation

This manuscript presents a well-designed quasi-experimental study with appropriate statistical analysis and a clearly described intervention. The topic is relevant and timely, particularly in the context of low- and middle-income healthcare systems. The revisions have effectively addressed previous concerns, and the manuscript now makes a meaningful contribution to the literature on evidence-based practice education among nurses.
